# Gliadin Peptide Facilitates FITC Dextran Transport across the Non Everted Gut Sac of Rat Small Intestine

**DOI:** 10.3390/scipharm86020013

**Published:** 2018-04-10

**Authors:** Ratna Annisa Utami, Aunillah Hakiki, Sukmadjaja Asyarie, Debbie Soefie Retnoningrum

**Affiliations:** Laboratory of Pharmaceutical Biotechnology, School of Pharmacy, Institut Teknologi Bandung, Jalan Ganesha No. 10 Bandung, West Java 40132, Indonesia; ratna@fa.itb.ac.id (R.A.U.); aunillahhakiki@gmail.com (A.H.); sukmada@fa.itb.ac.id (S.A.)

**Keywords:** FITC-Dextran, gliadin, permeation, rat intestine, SOD *Citrus limon*

## Abstract

Superoxide dismutase (SOD) is an antioxidant protein. When administered orally, it has low bioavailability due to its low permeation. In a previous study we fused gliadin peptide P51 (LGQQQPFPPQQPYPQPQPF) and gliadin peptide P61 (QQPYPQPQPF) with SOD *Citrus limon* (SOD_Cl), namely GliSOD_P51 and GliSOD_P61 to increase permeation of SOD_Cl through intestine. In this work, the permeation of fluorescein isothiocyanate (FITC)-Dextran 10 kDa, FD10 and 40 kDa, FD40 as paracellular transport markers across excised rat intestinal wall was investigated with the presence of GliSOD_P51 and GliSOD_P61. A permeability study was performed using non-everted rat intestine by incubating FD10 or FD40 with SOD_Cl, and GliSOD_P61. The presence of SOD_Cl, GliSOD_P51 or GliSOD_P61 inside intestine (apical) and outside intestine (basolateral) was analyzed by protein electrophoresis. The concentration of FD that penetrated to the basolateral solution was analyzed by spectrofluorometry. Sodium dodecyl sulfate polyacrylamide gel electrophoresis (SDS-PAGE) analysis revealed the presence of GliSOD_P51 and GliSOD_P61 but not SOD_Cl in basolateral compartment. The percentage of FD10 but not FD40 and SOD_Cl that penetrated to the basolateral solution significantly increased with the presence of gliadin in GliSOD_P51 and GliSOD_P61. GliSOD_P51 and GliSOD_P61 are able to penetrate the rat intestinal epithelial membrane and the gliadin peptides facilitate FD10 to penetrate the epithelial.

## 1. Introduction

Oxidative stress due to the higher generation of reactive oxygen species (ROS) is a prevalent condition in various diseases, including cancer, asthma, diabetes, arthritis, atherosclerosis, aging, infertility, neurological disorders, ischemia-reperfusion injury, transplant rejection, autoimmune diseases, rheumatoid arthritis, and septic shock-induced tissue injury [1,2]. Superoxidedismutase (SOD) is a primary antioxidant enzyme that plays an important role in scavenging the ROS. Due to this activity, this enzyme is considered as a potential therapeutic compound which is important in dealing with oxidative stress [3], and clinical studies of SOD in human subjects showed promising results [4,5]. 

Protein delivery via oral route has been a persistent challenge. Protein should have the ability to be efficiently absorbed from the gastrointestinal tract and to cross biological membranes. However, the low lipophilicity and large molecular mass of proteins limit their absorption [6,7,8]. Owing to its high molecular size and hydrophilicity in nature, SOD is difficult to absorb into the intestinal tract. 

Some evidence suggests that gliadin peptides can cross the intestinal barrier. Despite their history as the factor responsible for the development of intestinal damage in Coeliac Disease (CD) patients, some studies showed that gliadin peptides were not toxic. Gliadin-derived peptide (LGQQQPFPPQQPYPQPQPF) did not cause toxicity in healthy jejunal specimens, showing that the toxicity was specific to CD [9]. When recombinant Cu–Zn SOD from *Cucumis melo* or Cu–Zn SOD from *Citrus limon* was fused with gliadin peptide (QQPYPQPQPF), both proteins interestingly showed high cell viability during cytotoxicity assay in vitro even up to 24 h, suggesting that this fusion protein did not elicit a cytotoxic effect on intestinal epithelial cells [10,11]. Another study showed that the gliadin-based nanoparticle could be a powerful tool for delivery and controlled release of anticancer drugs [12]. These findings constitute an opportunity for further evaluating gliadin for pharmaceutical application and may be as a promising lead compound for the development of a novel permeation-enhancing agent.

In order to assess that possibility, we recently developed two gliadin-derived peptides as permeation enhancer, namely gliadin peptide P51 (LGQQQPFPPQQPYPQPQPF) and gliadin peptide P61 (QQPYPQPQPF). In a previous study, we constructed a fusion protein GliSOD_P51 and GliSOD_P61 comprising gliadin peptide P51 or P61 and SOD *Citrus limon* (SOD_Cl) as a protein model. GliSOD_P61 showed permeation capacity crossed Caco-2 cells monolayer [11], while GliSOD_P51 has not been studied for this ability.

For screening of a permeation enhancer, a high-throughput method for evaluating intestinal permeability is desired [13]. An in vivo experimental system or in vitro using Caco-2 cell monolayer is ideal for determining the permeability ability. However, its use is hindered by the high cost or long cultural period which is typically unfeasible in the early stages of discovery of permeation enhancer candidate. In this study, we evaluated the permeation capacity and the effect of SOD_Cl, GliSOD_P51 and GliSOD_P61 on the paracellular transport of fluorescein isothiocyanate (FITC)-Dextran in rat small intestine using the in vitro non everted rat intestine sac model. We used this method since it has been widely used to predict in vivo absorption of drugs in humans with low cost and greater simplicity [14,15]. 

## 2. Materials and Methods

*Escherichia coli* BL21(DE3) (Invitrogen, Carlsbad, CA, USA) is maintained at the Laboratory of Pharmaceutical Biotechnology, School of Pharmacy, Institute of Technology Bandung and used for intracellular production of SOD_Cl, GliSOD_P51 and GliSOD_P61, respectively. The plasmids carrying DNA encoding SOD_Cl, GliSOD_P51 and GliSOD_P61 were each constructed in our previous study [16]. To create GliSOD_P51 or GliSOD_P61 fusion protein, gliadin peptide sequence P51 (LGQQQPFPPQQPYPQPQPF) and P61 (QQPYPQPQPF) was individually fused to the amino terminus of a complete amino acid sequence of SOD_Cl (accession No. AF318938, GenBank, NCBI). The amino acid sequence of P51 and P61 were obtained from sequence of alfa gliadin (*Triticum monococcum*) (accession No. ACJ76942.1, GenBank, NCBI).

### 2.1. Animals

Male Wistar rats (240–270 g), obtained from the School of Pharmacy, Institut Teknologi Bandung are maintained in a controlled environment of 25 °C with a 12–12 h light/dark cycle. The rats were fasted overnight before experimentation and had access to water ad libitum. All protocols and procedures experiments involving animal research plans were approved by the Animal Research Ethics Committee, School of Pharmacy, Institut Teknologi Bandung (Certificate No. 03/KEPHP-ITB/11-2016).

### 2.2. Production of SOD_Cl and GliSOD_P61

Overproduction and purification of SOD_Cl, GliSOD_P51 and GliSOD_P61 were performed according to a previously described procedure [16]. A single colony of *E. coli* strain BL21(DE3) containing each pET_16b_SOD_Cl, pET_16b_GliSOD_P51 and pJExpress416_GliSOD_P61 plasmids was inoculated into 5 mL Terrific Broth (TB) supplemented with appropriate antibiotics, respectively. An overnight culture (5%) of recombinant *E. coli* was used to inoculate 200 mL medium and was cultured to final OD600 of 0.6–0.8 at 37 °C with vigorous aeration (200 rpm). For protein overproduction, *E. coli* pJExpress416_GliSOD_P61 was induced by Isopropyl-β-d-thiogalactoside (IPTG) final concentration of 0.5 mM for 4 h. While for SOD_Cl and GliSOD_P51 production, the respective recombinant *E. coli* was cultured overnight (24 h) at 22 °C with vigorous aeration (200 rpm) without the addition of IPTG. 

The cell pellets were harvested by cold centrifugation (4500× *g*, 4 °C, 20 min), washed and resuspended 1:4 in Lysis-Equilibration-Wash (LEW) buffer (50 mM NaH_2_PO_4_, 300 mM NaCl, pH 8.0) containing 1 mM phenylmethylsulfonyl fluoride. The cell pellet was lysed by sonication using Ultrasonic Homogenizers CY-500 (Optic Ivymen System, Biotech SL, Madrid, Spain). Afterward, the cell debris and other insoluble fraction were separated from the crude extract by cold centrifugation for 20 min at 4500× *g.* The SOD proteins were individually purified using cOmplete™ His-tag Purification Resin (Roche Applied Science, Mannheim, Germany) from crude extract and eluted by 250 mM imidazole. All fractions containing purified protein were each concentrated using a 10 kDa Nanosep devices by cold centrifugation. The concentrated purified proteins were analyzed by a Coomassie brilliant blue stained 10% sodium dodecyl sulfate polyacrylamide gel electrophoresis (SDS-PAGE). 

### 2.3. In Vitro Studies Using the Non-Everted Gut Sac Method

The ability of recombinant proteins crossing intestine walls was determined using the non-everted gut sac method followed by the published methods with slight modification [17]. The non-everted gut sac method involves placing the sample solution in the rat gut sac without everting. The non-everted gut sac was prepared by quickly removing the small intestine from starved rats killed under CO_2_-anesthesia. The intestine was excised (±3 cm) and rinsed with Ringer Lactate Solution at room temperature. The jejunum was knotted at one end, filled with sample solutions and the other end was knotted. This intestine bag (regarded as apical compartment) was placed in an acceptor (basolateral compartment) solution LEW buffer for 2 h at 37 °C, 50 rpm. After 2 h, the remaining samples in the apical and basolateral compartments were independently collected and their protein contents were each evaluated by an SDS-PAGE analysis with coomasie blue staining. The FD10 or FD40 concentration was quantified from the solutions from basolateral compartment using Spectrofluorometer Shimadzu RF-5301 PC (excitation wavelength, 488 nm; detection wavelength, 521 nm). Samples used in these experiments were SOD_Cl, GliSOD_P51 or GliSOD_P61 (40 or 200 µg) with or without FD10 or FD40 (100 µg). 

## 3. Results

### 3.1. Proteins Production

**Figure 1:**
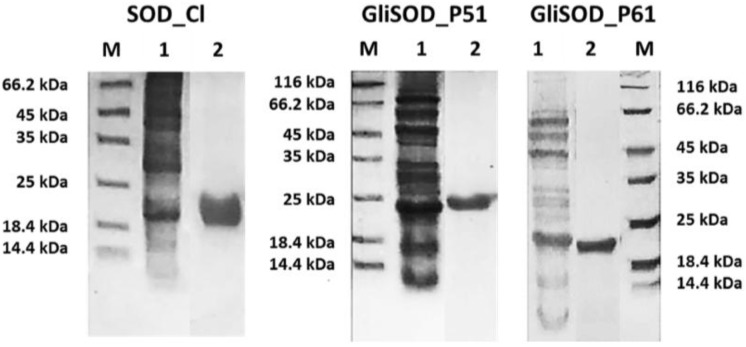
Sodium dodecyl sulfate polyacrylamide gel electrophoresis (SDS-PAGE) analysis of crude and purified proteins; M = protein molecular weight marker,1 = crude protein; 2 = purified protein. SOD_Cl = Superoxide dismutase *Citrus limon*; GliSOD_P51: SOD_Cl-Gliadin P51; GliSOD_P61: SOD_Cl-Gliadin P61.

The purified proteins were each concentrated and dialyzed against LEW Buffer to remove the imidazole. The overproduction system and one step purification using Nickel affinity column was sufficient to obtain a sufficient amount of pure protein (Figure 1).

### 3.2. Permeability Assay

**Figure 2:**
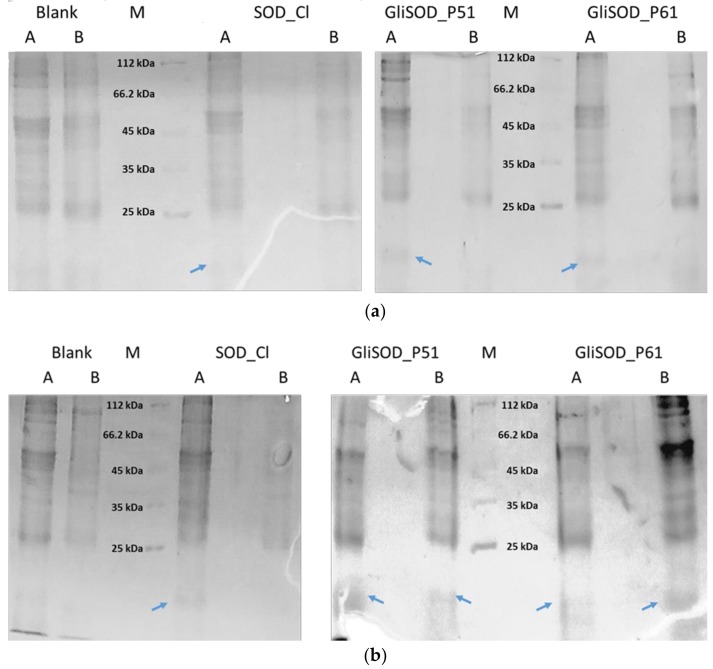
Electrophoregram of proteins from the apical and basolateral solutions collected after non-everted gut sac incubation with 40 µg (**a**) or 200 µg (**b**) of SOD_Cl, GliSOD_P51 or GliSOD_P61. A = the content of the apical solution and B = the content of basolateral solution. Blue arrows show protein bands of SOD_Cl, GliSOD_P51 or GliSOD_P61, respectively.

In this study, we further confirmed the permeation capacity of GliSOD_P61, investigated the role of gliadin peptide P51 in GliSOD_P51 as a permeation enhancer and assessed its potential for the oral delivery of SOD_Cl. In a permeability assay, we first used 40 µg proteins followed the amount of proteins used in our previous publication [11]. However, after 2 h incubation, the results of protein electrophoresis showed there was no GliSOD_P51, GliSOD_P61 or SOD_Cl band in the basolateral compartment (Figure 2a). In the next experiment, we increased the protein amount in the permeability assay. After 2 h incubation using 200 µg proteins, we observed indistinct bands smaller than 25 kDa in size in SDS-PAGE gels in basolateral compartment solution of GliSOD_P51 and GliSOD_P61 but no corresponding protein band of SOD_Cl in basolateral compartment solution (Figure 2b). These smaller and indisctinct bands were absent in the control solution, suggesting that they originated from the GliSOD_P51 or GliSOD_P61 solution in the apical compartment. This indicated the success of gliadin peptide of GliSOD_P51 or GliSOD_P61 to enable the SODs to pass through the intestinal cell barrier. These results were in line with our previous permeability assay in Caco-2 cell monolayer, where GliSOD_P61 but not SOD_Cl penetrated Caco-2 cell monolayer [11]. In this report, we demonstrated that the presence of LGQQQPFPPQQPYPQPQPF and QQPYPQPQPF gliadin peptides in SOD_Cl enabled the SODs to be readily absorbed from the non-everted intestine of the rat.

### 3.3. Paracellular Permeability Assay

To investigate whether gliadins in GliSOD_P51 and GliSOD_P61 were responsible for protein transport via the paracelluar route, the cells were co-incubated with both SODs and FITC-Dextran. FITC-Dextran is the marker used to study the paracelluar absorption along the small intestine [18]. To evaluate the size selectivity of the paracellular pathway, we used FITC-Dextran with two different sizes, 10 kDa and 40 kDa. The effect of the presence of GliSOD_P51 and GliSOD_P61 on FD10 and FD40 transport was evaluated. Our results showed that there was no significant effect on the permeability of FD10 observed in the presence and absence of 40 µg GliSOD_P51 and GliSOD_P61 compared to SOD_Cl in the same amount (3.00 ± 0.95%; 3.23 ± 0.16% and 3.3 ± 1.3%, respectively). GliSOD_P51 and GliSOD_P61 in the same amount also did not affect the absorption of FD40 (data not shown). This indicated that neither of the 40 µg SODs increased the permeation of FD10 or FD40. 

**Figure 3:**
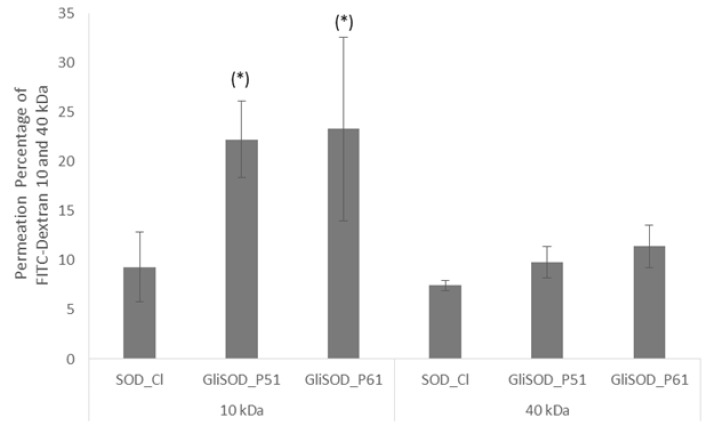
Effect of GliSOD_P51 and GliSOD_P61 on the permeability for paracellular markers FD10 and FD40. Paracellular marker flux measurement revealed significant effect on the permeability of molecules to 10 kDa in size when cells were incubated with GliSOD_P51 and GliSOD_P61 compared to SOD_Cl. The passage of FD40 was not significantly changed. Data were expressed as mean ± standard deviation (*n* = 3). (*) indicates significant difference (*p* < 0.05) compared to SOD_Cl.

When the amount of protein was increased (200 µg), GliSOD_P51 and GliSOD_P61 significantly affected the permeability of FD10 compared to the SOD_Cl (*p* > 0.05). However, the same amount of GliSOD_P51 and GliSOD_P61 did not affect FD40 absorption. The percentage of permeation of both FITC-Dextran after incubation with SOD_Cl, GliSOD_P51 and GliSOD_P61 is depicted in Figure 3.

## 4. Discussion

In our previous study, GliSOD_P61 but not SOD_Cl was found to permeate through the Caco-2 cell monolayer via a mechanism involving tight junctions opening without affecting the *ZO-1* gene expression and no toxicity effect [11] was observed demonstrating that gliadin peptide P61 had potential as a permeation enhancer. In this study, we further confirmed the permeation capacity of GliSOD_P61 and also examined the capacity of P51 as a permeation enhancer for SOD_Cl. The gliadin peptide LGQQQPFPPQQPYPQPQPF was found to penetrate the intestine after oral administration in mice [19]. This peptide was also transported in intact form after incubation in using chambers across duodenal biopsy specimens from patients with Celiac disease [20]. 

We used non-everted rat intestine for the permeability study. Non-everted rat intestine methods were used since the permeability assay using Caco-2 cells monolayer requires a long culturing period of at least 21 days. Hence, this limits the throughput and usefulness, especially for permeation enhancer fast screening. Permeability assay was carried out within the first hour after the intestine was collected to ensure that the cells had viability and activity [17]. The results from this study showed that all SODs except SOD_Cl were transported across the non-everted rat intestine. These results were in agreement with our previous GliSOD_P61 permeability assay in Caco-2 cell monolayer, demonstrating that P61 accounted for the transport of SOD_Cl across the intestinal barrier [19,20]. The SOD concentrations used in the non-everted rat intestine method was higher than the concentration used in the Caco2 cell monolayer method. The difference in concentration may be attributed to the larger intestine surface in the non-everted rat intestine compared to the Caco2 cell monolayer. 

In the present study, we demonstrated that, in the presence of gliadin, permeability of FD 10 but not FD40 across intestine was increased. The increase of FD10 transport when co-incubated with GliSOD_P51 and GliSOD_P61 indicated that both gliadin peptides could regulate TJ opening or paracellular pathway. This finding is in agreement with the study by Lammers and coworkers that reported the permeability enhancement by gliadin intact protein through opening of tight junction [21]. Interestingly, GliSOD_P51 and GliSOD_P61 did not increase the FD40 transport, indicating that tight junction opening did not permit the passage of larger molecules size equal to the size of FD40. Our result strongly indicated that the effect of gliadin to the permeation via tight junction opening was size-selective. However, SDS-PAGE analysis revealed that gliadin peptides can also facilitate the transport protein, indicating that opening tight junction results may have been related to different sizes and conformations. It is known that dextrans are unbranched polyglucans and FDs present a linear conformation [22] while proteins are folded into compact structure. This phenomenon is in line with several studies showing that sodium caprate and C-terminal fragment *Clostridium perfringens* enterotoxin (C-CPE) as permeation enhancer was also size-selective. Sodium caprate increased the permeation of FITC-Dexran 4 and 20 kDa through Caco-2 cells and C-CPE enhanced that of FITC-Dextran 4 and 10 kDa permeation but not FITC-Dextran 20 and 40 kDa through intestine [23]. 

A critical issue in the clinical application of permeation enhancer is safety. This includes the safety of gliadin peptide in itself and that of the modulation of TJ, i.e., the entry of unwanted substances by the opening of TJs [24]. The gliadin safety is still being debated and, as mentioned above, several studies showed toxicity of gliadin peptide but others claimed no toxicity in several cell lines or healthy tissue [9,10,11,12]. Although further detailed analyses are necessary to understand the involvement of TJs in the enhancement of absorption by gliadin peptide, the present findings hypothesize that gliadin P51 and P61 can facilitate the opening of TJs and display activity as an absorption enhancer. However, there remain challenges to be overcome such as the potential of immunogenicity and toxicity of these peptides because permeation enhancer would be used as additive in formulation and repeatedly administered.

## 5. Conclusions

Taken together, the results of this study demonstrate the activity of gliadin peptides, P51 and P61 as permeation enhancer and their potency in oral protein delivery. However, further research in a preclinical setting is necessary to provide evidence for their safety and efficacy and to disclose their precise molecular mechanism in increasing paracellular transport. 

The use of gliadin-derived peptide opens new perspectives as a biomaterial for permeation enhancer in a drug delivery system. Here, we also report that the use of the non-everted gut sac method in permeability assay for screening of permeation enhancer showed similar results to the Caco-2 cell method.
